# Supplementary material for: Fruit quality and antioxidant potential of Prunus humilis Bunge accessions
Source: PLoS One. 2020 Dec 30;15(12):e0244445. doi: 10.1371/journal.pone.0244445 (PMC7773198; doi:10.1371/journal.pone.0244445)
Supplement: S1 Fig — (DOC) [file pone.0244445.s001.doc]

Fig.S1 Cluster analysis of fruit flavonoid content in 137 *Prunus humilis* accessions
