# Supplementary material for: Fruit quality and antioxidant potential of Prunus humilis Bunge accessions
Source: PLoS One. 2020 Dec 30;15(12):e0244445. doi: 10.1371/journal.pone.0244445 (PMC7773198; doi:10.1371/journal.pone.0244445)
Supplement: S1 Table — (DOC) [file pone.0244445.s002.doc]

Supplement 1. The background, maturing period and peel color of *Cerasus humilis* accessions

| Accessions | Peel color | Maturing period | Background |
| --- | --- | --- | --- |
| 3-17-4 | Red | Late Jul | Seed Selection |
| JD1-6-7-37 | Red | Late Jul | Seed Selection |
| 10-02 | Red-orange | Late Jul | Seed Selection |
| 3-17-2 | Red | Late Jul | Seed Selection |
| 3-60-2-8 | Light red | Late Jul | Seed Selection |
| HB-2 | Red | Late Jul | Seed Selection |
| HB-1 | Red | Late Jul | Seed Selection |
| 3-52-2-14D | Red-orange | Late Jul | Seed Selection |
| 3-55-HY | Yellow | Late Jul | Seed Selection |
| 3-17-5-1 | Red | Late Jul | Seed Selection |
| GLG | Yellow | Late Jul | Seed Selection |
| TWS | Red | Mid-Sep | Seed Selection |
| K1 | Red | Late Jul | Seed Selection |
| Fh-1 | Red | Early Aug | Seed Selection |
| Y09-15 | Red | Late Aug | Seed Selection |
| 08-33N | Red | Late Jul | Seed Selection |
| HPOL | Red-orange | Late Aug | Seed Selection |
| 3-17-5-2 | Red | Late Jul | Seed Selection |
| 16-14 | Red | Early Aug | Seed Selection |
| TKQCB | Red | Early Sep | Seed Selection |
| DG-6 | Red | Late Jul | Seed Selection |
| SG | Red | Late Jul | Seed Selection |
| 3-17-5 | Red-orange | Late Jul | Seed Selection |
| 99-02 | Red-orange | Late Aug | Seed Selection |
| 10-21 | Red-orange | Mid-Aug | Seed Selection |
| DG-41 | Red | Mid-Aug | Seed Selection |
| 19-09 | Red-orange | Late Jul | Seed Selection |
| T1-5-17-1 | Red-orange | Early Sep | Seed Selection |
| 3-21-1-2 | Red | Late Aug | Seed Selection |
| YS1HXT | Red | Late Jul | Introduction |
| T-HB-11 | Red | Mid-Aug | Seed Selection |
| TB17-1 | Yellow | Late Jul | Seed Selection |
| 15-12 | Red | Mid-Aug | Seed Selection |
| 11-20-1 | Red | Mid-Aug | Seed Selection |
| 5N17-2 | Red | Late Jul | Seed Selection |
| Y04-27 | Yellow | Early Aug | Seed Selection |
| 12-3 | Red | Late Jul | Seed Selection |
| 08-16 | Yellow | Late Aug | Seed Selection |
| XF-1 | Red | Early Aug | Seed Selection |
| HY-1 | Red | Late Jul | Seed Selection |
| M13-2 | Red | Late Jul | Seed Selection |
| XZ-2 | Light red | Mid-Jul | Seed Selection |
| 02-17 | Light red | Mid-Aug | Seed Selection |
| Y14-26 | Red | Early Aug | Seed Selection |
| JO-1 | Dark red | Mid-Jul | Seed Selection |
| GZ | Red | Late Jul | Seed Selection |
| 19-04 | Red | Early Sep | Seed Selection |
| JO-2 | Dark red | Mid-Jul | Seed Selection |
| Y07-14 | Red-orange | Late Jul | Seed Selection |
| JO2H | Red | Late Jul | Introduction |
| 19-06 | Red | Late Jul | Seed Selection |
| 01-01 | Red-orange | Late Aug | Seed Selection |
| 19-03 | Red-orange | Late Aug | Seed Selection |
| Y05-17 | Red-orange | Late Aug | Seed Selection |
| X17-01 | Red | Late Jul | Seed Selection |
| 03-38 | Red-orange | Late Jul | Seed Selection |
| 03-25 | Red | Mid-Jul | Seed Selection |
| T-HB-3 | Red-orange | Late Jul | Seed Selection |
| T1-10-17-2 | Red | Mid-Aug | Seed Selection |
| 19-07 | Red-orange | Late Aug | Seed Selection |
| YYZHFBH | Red-orange | Early Aug | Seed Selection |
| 15-02 | Red | Late Jul | Seed Selection |
| 3-40-1-1 | Red | Late Aug | Seed Selection |
| 3-29-3-2 | Red-orange | Early Aug | Seed Selection |
| 09-19 | Yellow | Early Sep | Seed Selection |
| 11-20M | Red | Late Jul | Seed Selection |
| T-HB-10 | Red | Mid-Aug | Seed Selection |
| JHY | Red | Late Jul | Seed Selection |
| 10-33 | Red | Mid-Aug | Seed Selection |
| Y08-22 | Red | Early Aug | Seed Selection |
| HB-5 | Red-orange | Late Jul | Seed Selection |
| Y03-10 | Red | Mid-Sep | Seed Selection |
| K2 | Yellow | Late Aug | Seed Selection |
| 09-03 | Red | Early Oct | Seed Selection |
| 10-06 | Yellow | Early Aug | Seed Selection |
| 3-5-1-14 | Red | Early Aug | Seed Selection |
| 15-40 | Yellow | Mid-Aug | Seed Selection |
| DG-7 | Red | Late Jul | Seed Selection |
| JO1H | Red | Late Jul | Introduction |
| F3-1 | Red-orange | Late Jul | Seed Selection |
| 09-38-1 | Red | Late Jul | Seed Selection |
| J-2 | Yellow | Mid-Jul | Seed Selection |
| DG-4-1 | Yellow | Late Jul | Seed Selection |
| PZBG | Yellow | Mid-Aug | Seed Selection |
| Ft3-1-2 | Red | Early Aug | Seed Selection |
| S-D-2 | Red | Early Aug | Seed Selection |
| 15-01 | Red-orange | Late Jul | Seed Selection |
| N15-42 | Red | Late Aug | Seed Selection |
| TXG | Red | Late Jul | Seed Selection |
| SGX | Red | Early Aug | Seed Selection |
| 1-17-2 | Red | Mid-Aug | Seed Selection |
| 11-07 | Dark red | Late Jun | Seed Selection |
| M19-4 | Red | Mid-Jul | Seed Selection |
| Ft-4 | Red | Early Sep | Seed Selection |
| GS-2 | Red | Early Aug | Seed Selection |
| 628-1 | Red | Mid-Aug | Seed Selection |
| Y06-22 | Red-orange | Early Sep | Seed Selection |
| S-D-3 | Yellow | Early Aug | Seed Selection |
| 16-11 | Red | Early Aug | Seed Selection |
| 3-17-1 | Red | Mid-Jul | Seed Selection |
| DS-1 | Light red | Mid-Jul | Seed Selection |
| Y13-03 | Red-orange | Early Aug | Seed Selection |
| 5B17-3 | Red | Late Jul | Seed Selection |
| 5N17-2-1 | Red | Late Jul | Seed Selection |
| 10-04 | Yellow | Mid-Aug | Seed Selection |
| 34-1 | Red | Late Jul | Seed Selection |
| Ft3-1-1 | Red | Early Aug | Seed Selection |
| 03-35 | Red | Early Aug | Seed Selection |
| 02-14 | Yellow | Mid-Aug | Seed Selection |
| 13-05 | Red-orange | Late Jul | Seed Selection |
| 15-10 | Red | Mid-Aug | Seed Selection |
| M19-4-1 | Light red | Late Jul | Seed Selection |
| 15-42 | Light red | Late Aug | Seed Selection |
| 3-30-17-1 | Yellow | Early Sep | Seed Selection |
| ZYOL | Dark red | Mid-Aug | Seed Selection |
| XZ-1 | Red | Mid-Jul | Seed Selection |
| 16-10M | Light red | Mid-Jul | Seed Selection |
| HB-8 | Red | Mid-Aug | Seed Selection |
| 09-38-2 | Red-orange | Late Jul | Seed Selection |
| FDHG | Red | Early Aug | Seed Selection |
| 19-05 | Yellow | Mid-Aug | Seed Selection |
| Ft-1 | Red | Late Jul | Seed Selection |
| 10-03 | Red | Late Aug | Seed Selection |
| 3-4-4-20 | Red | Late Aug | Seed Selection |
| LB-28 | Yellow | Mid-Aug | Seed Selection |
| Y13-09 | Yellow | Early Sep | Seed Selection |
| T-HB-4 | Red | Late Jul | Seed Selection |
| S-D-1 | Red | Early Aug | Seed Selection |
| 15-51 | Red | Late Jul | Seed Selection |
| 15-11 | Red | Mid-Aug | Seed Selection |
| 628 | Dark red | Late Jun | Seed Selection |
| 08-24 | Red | Mid-Jul | Seed Selection |
| HB-6 | Yellow | Late Jul | Seed Selection |
| T1-1-17-1 | Red | Mid-Aug | Seed Selection |
| 3-3-4-5 | Red | Late Aug | Seed Selection |
| T17-1 | Red | Late Jul | Seed Selection |
| ZS-3 | Red | Mid-Aug | Seed Selection |
