# Supplementary material for: Fruit quality and antioxidant potential of Prunus humilis Bunge accessions
Source: PLoS One. 2020 Dec 30;15(12):e0244445. doi: 10.1371/journal.pone.0244445 (PMC7773198; doi:10.1371/journal.pone.0244445)
Supplement: S2 Table — (DOC) [file pone.0244445.s003.doc]

Supplement 2. A list of abbreviation

| Abbreviation | Full Name |
| --- | --- |
| TFC | Total flavonoid content |
| TPC | Total phenol content |
| DPPH | 1,1-diphenyl-2-picrylhydrazyl |
| ABTS | 2,2’-azino-bis(3-ethylbenzothiazoline-6-sulfonic acid) |
| TPTZ | 2,4,6-tri-(2-pyridyl)-1,3,5-triazine |
| SSC | Soluble solid content |
| FRAP | Ferric reducing antioxidant power |
| CC | Catechin |
| EC | Epicatechin |
| LR | Liquiritigenin |
| RT | Rutin |
| Q3G | Quercetin-7-O-β-D-glucopyranoside |
| C3G | Cyanidin-3-O-glucoside |
| PCA | Principal component analysis |
