# Supplementary material for: Fruit quality and antioxidant potential of Prunus humilis Bunge accessions
Source: PLoS One. 2020 Dec 30;15(12):e0244445. doi: 10.1371/journal.pone.0244445 (PMC7773198; doi:10.1371/journal.pone.0244445)
Supplement: S3 Table — (DOC) [file pone.0244445.s004.doc]

| Accessions | Peel color | Single fruit weight (g) | Stone weight (g) | Vertical diameter (mm) | Horizontal diameter (mm) | Soluble solid content (%) | Total flavonoid content (mg/g FW) | Total phenol content (mg/g FW) | DPPH (mg TE/g FW) | FRAP (mg TE/g FW) | ABTS (mg TE/g FW) |
| --- | --- | --- | --- | --- | --- | --- | --- | --- | --- | --- | --- |
| 3-17-4 | Red | 3.55 | 0.42 | 17.96 | 18.92 | 17.62 | 28.37 | 9.02 | 6.21 | 13.64 | 24.23 |
| JD1-6-7-37 | Red | 3.45 | 0.64 | 18.42 | 18.61 | 13.20 | 21.99 | 7.72 | 8.19 | 16.82 | 22.63 |
| 10-02 | Red-orange | 6.73 | 0.60 | 20.83 | 23.98 | 16.33 | 20.45 | 6.72 | 5.03 | 10.00 | 18.81 |
| 3-17-2 | Red | 5.86 | 0.46 | 19.60 | 21.28 | 16.28 | 19.20 | 6.61 | 7.39 | 13.38 | 20.26 |
| 3-60-2-8 | Light red | 3.49 | 0.46 | 18.01 | 18.00 | 13.83 | 18.52 | 7.15 | 6.25 | 13.50 | 18.78 |
| HB-2 | Red | 2.22 | 0.24 | 15.39 | 16.29 | 15.04 | 18.19 | 6.56 | 5.26 | 13.47 | 19.47 |
| HB-1 | Red | 3.48 | 0.34 | 16.57 | 18.49 | 15.10 | 17.47 | 6.34 | 7.00 | 12.99 | 17.93 |
| 3-52-2-14D | Red-orange | 3.64 | 0.42 | 17.73 | 18.22 | 15.08 | 17.30 | 6.67 | 6.04 | 12.04 | 17.89 |
| 3-55-HY | Yellow | 12.23 | 0.41 | 20.08 | 18.22 | 15.85 | 16.07 | 6.21 | 6.59 | 12.37 | 17.32 |
| 3-17-5-1 | Red | 4.46 | 0.30 | 16.75 | 19.39 | 15.08 | 16.01 | 6.46 | 5.70 | 12.24 | 16.77 |
| GLG | Yellow | 6.29 | 0.45 | 25.54 | 21.03 | 14.60 | 15.96 | 5.56 | 4.26 | 9.00 | 15.47 |
| TWS | Red | 3.51 | 0.40 | 18.78 | 19.18 | 15.60 | 15.23 | 5.41 | 5.95 | 12.03 | 16.75 |
| K1 | Red | 3.66 | 0.40 | 16.41 | 21.08 | 13.82 | 15.07 | 5.31 | 5.63 | 11.31 | 19.51 |
| Fh-1 | Red | 9.76 | 0.94 | 19.67 | 27.03 | 14.08 | 15.05 | 5.44 | 5.76 | 11.88 | 21.41 |
| Y09-15 | Red | 8.84 | 0.40 | 22.55 | 27.53 | 12.18 | 15.05 | 5.42 | 6.40 | 10.78 | 15.64 |
| 08-33N | Red | 4.78 | 0.58 | 19.39 | 20.54 | 12.43 | 14.96 | 5.71 | 6.73 | 11.26 | 17.09 |
| HPOL | Red-orange | 2.89 | 0.32 | 17.18 | 17.31 | 12.24 | 14.80 | 5.65 | 6.36 | 11.74 | 16.66 |
| 3-17-5-2 | Red | 3.98 | 0.35 | 16.91 | 18.54 | 17.03 | 14.72 | 5.69 | 6.67 | 11.83 | 16.98 |
| 16-14 | Red | 6.00 | 0.77 | 21.23 | 20.71 | 14.58 | 14.65 | 4.72 | 4.60 | 10.20 | 15.45 |
| TKQCB | Red | 2.91 | 0.35 | 14.51 | 17.11 | 19.40 | 14.57 | 4.62 | 5.67 | 10.60 | 16.55 |
| DG-6 | Red | 3.95 | 0.31 | 17.01 | 18.95 | 14.90 | 14.36 | 5.10 | 6.15 | 10.90 | 16.12 |
| SG | Red | 6.63 | 0.56 | 22.54 | 22.84 | 13.20 | 14.03 | 5.36 | 6.25 | 10.74 | 15.94 |
| 3-17-5 | Red-orange | 4.24 | 0.29 | 17.95 | 18.29 | 13.50 | 13.90 | 5.40 | 5.64 | 11.43 | 20.66 |
| 99-02 | Red-orange | 3.09 | 0.29 | 16.39 | 17.60 | 20.36 | 13.88 | 5.73 | 5.05 | 11.78 | 16.21 |
| 10-21 | Red-orange | 6.13 | 0.41 | 20.19 | 22.89 | 17.77 | 13.74 | 3.55 | 5.00 | 10.12 | 15.26 |
| DG-41 | Red | 9.74 | 0.81 | 22.63 | 26.20 | 14.45 | 13.43 | 4.48 | 6.04 | 9.43 | 13.95 |
| 19-09 | Red-orange | 9.46 | 0.86 | 23.31 | 26.46 | 13.30 | 13.41 | 4.31 | 6.03 | 8.88 | 13.72 |
| T1-5-17-1 | Red-orange | 2.94 | 0.30 | 17.23 | 17.59 | 19.86 | 13.37 | 5.36 | 4.80 | 10.74 | 15.25 |
| 3-21-1-2 | Red | 3.03 | 0.35 | 16.58 | 18.20 | 16.68 | 13.30 | 5.15 | 4.47 | 9.64 | 15.29 |
| YS1HXT | Red | 1.20 | 0.17 | 12.13 | 12.97 | 15.25 | 13.14 | 5.28 | 5.54 | 11.11 | 15.88 |
| T-HB-11 | Red | 9.70 | 0.61 | 23.61 | 26.07 | 14.70 | 13.03 | 4.35 | 5.37 | 9.69 | 14.96 |
| TB17-1 | Yellow | 10.12 | 0.59 | 25.67 | 27.20 | 15.23 | 12.94 | 4.74 | 3.79 | 7.81 | 13.58 |
| 15-12 | Red | 7.85 | 0.56 | 21.50 | 25.06 | 15.33 | 12.91 | 4.72 | 5.71 | 9.11 | 14.77 |
| 11-20-1 | Red | 5.76 | 0.35 | 19.18 | 22.83 | 17.00 | 12.84 | 4.82 | 4.71 | 9.34 | 14.35 |
| 5N17-2 | Red | 4.36 | 0.25 | 17.40 | 21.06 | 13.70 | 12.78 | 4.64 | 4.97 | 9.89 | 14.40 |
| Y04-27 | Yellow | 5.47 | 0.44 | 18.12 | 22.74 | 8.50 | 12.71 | 4.63 | 4.84 | 10.03 | 14.35 |
| 12-3 | Red | 1.20 | 0.18 | 13.95 | 12.84 | 12.38 | 12.70 | 4.82 | 4.54 | 9.91 | 14.30 |
| 08-16 | Yellow | 9.63 | 0.66 | 26.41 | 25.30 | 11.38 | 12.55 | 4.70 | 4.48 | 9.72 | 14.47 |
| XF-1 | Red | 8.35 | 0.37 | 21.76 | 24.01 | 12.64 | 12.43 | 5.06 | 5.34 | 11.14 | 18.73 |
| HY-1 | Red | 8.59 | 0.67 | 24.02 | 25.99 | 13.50 | 12.40 | 4.90 | 4.82 | 9.50 | 18.76 |
| M13-2 | Red | 2.22 | 0.21 | 16.90 | 15.56 | 16.78 | 12.24 | 4.33 | 5.56 | 9.81 | 14.67 |
| XZ-2 | Light red | 2.53 | 0.30 | 15.26 | 17.30 | 8.64 | 12.21 | 4.20 | 4.45 | 8.45 | 12.43 |
| 02-17 | Light red | 7.97 | 0.51 | 22.37 | 25.04 | 15.17 | 12.13 | 5.05 | 4.93 | 9.79 | 13.13 |
| Y14-26 | Red | 10.50 | 0.69 | 24.69 | 25.75 | 9.48 | 12.06 | 4.50 | 5.35 | 10.10 | 17.09 |
| JO-1 | Dark red | 3.01 | 0.29 | 15.51 | 17.79 | 9.18 | 11.93 | 4.67 | 4.71 | 9.86 | 13.71 |
| GZ | Red | 7.94 | 0.49 | 22.19 | 24.71 | 14.77 | 11.90 | 4.76 | 4.79 | 9.91 | 17.85 |
| 19-04 | Red | 6.47 | 0.41 | 18.76 | 22.77 | 16.00 | 11.65 | 4.36 | 5.55 | 9.89 | 15.09 |
| JO-2 | Dark red | 2.58 | 0.29 | 15.05 | 17.33 | 10.27 | 11.60 | 4.56 | 4.35 | 10.46 | 14.18 |
| Y07-14 | Red-orange | 7.55 | 0.72 | 20.64 | 23.29 | 14.60 | 11.48 | 4.28 | 5.23 | 9.43 | 16.51 |
| JO2H | Red | 3.78 | 0.29 | 17.69 | 19.21 | 14.24 | 11.20 | 4.49 | 5.35 | 9.21 | 12.81 |
| 19-06 | Red | 8.15 | 0.50 | 22.56 | 25.03 | 15.96 | 11.08 | 4.26 | 5.13 | 9.51 | 14.10 |
| 01-01 | Red-orange | 7.70 | 0.49 | 23.44 | 23.93 | 12.62 | 11.07 | 4.15 | 4.08 | 7.91 | 11.42 |
| 19-03 | Red-orange | 5.26 | 0.44 | 20.79 | 21.81 | 13.96 | 11.02 | 4.53 | 5.55 | 8.87 | 14.11 |
| Y05-17 | Red-orange | 4.19 | 0.25 | 17.41 | 20.37 | 17.83 | 10.99 | 4.38 | 5.60 | 9.19 | 14.04 |
| X17-01 | Red | 6.53 | 0.48 | 18.90 | 22.68 | 11.33 | 10.96 | 3.84 | 3.94 | 8.53 | 12.95 |
| 03-38 | Red-orange | 4.34 | 0.45 | 16.70 | 19.48 | 7.04 | 10.93 | 3.95 | 4.92 | 8.79 | 12.48 |
| 03-25 | Red | 3.15 | 0.30 | 17.32 | 18.17 | 13.92 | 10.75 | 4.09 | 4.32 | 8.14 | 15.23 |
| T-HB-3 | Red-orange | 6.54 | 0.38 | 20.97 | 23.16 | 15.68 | 10.57 | 3.83 | 4.23 | 7.75 | 11.71 |
| T1-10-17-2 | Red | 5.74 | 0.40 | 19.82 | 22.21 | 17.42 | 10.52 | 3.74 | 3.83 | 7.61 | 11.61 |
| 19-07 | Red-orange | 10.43 | 0.50 | 21.83 | 28.32 | 11.50 | 10.45 | 2.79 | 5.21 | 7.30 | 10.95 |
| YYZHFBH | Red-orange | 8.68 | 0.95 | 23.33 | 25.10 | 15.52 | 10.44 | 4.36 | 3.59 | 7.09 | 9.39 |
| 15-02 | Red | 4.35 | 0.48 | 18.96 | 20.35 | 16.56 | 10.42 | 3.89 | 4.82 | 7.73 | 11.54 |
| 3-40-1-1 | Red | 5.88 | 0.38 | 19.54 | 22.33 | 18.50 | 10.24 | 3.62 | 3.43 | 7.37 | 11.61 |
| 3-29-3-2 | Red-orange | 5.16 | 0.45 | 20.21 | 20.29 | 13.52 | 10.18 | 3.91 | 4.57 | 7.07 | 12.33 |
| 09-19 | Yellow | 6.78 | 0.64 | 23.98 | 22.18 | 17.20 | 10.11 | 4.00 | 4.96 | 8.35 | 12.51 |
| 11-20M | Red | 2.13 | 0.25 | 14.95 | 14.89 | 15.42 | 10.11 | 4.49 | 5.22 | 8.58 | 13.86 |
| T-HB-10 | Red | 7.69 | 0.37 | 20.88 | 25.70 | 16.23 | 10.10 | 3.55 | 3.55 | 7.26 | 11.34 |
| JHY | Red | 4.52 | 0.39 | 20.88 | 21.23 | 17.82 | 9.93 | 3.32 | 3.45 | 6.76 | 10.48 |
| 10-33 | Red | 8.48 | 0.61 | 18.75 | 26.51 | 14.93 | 9.92 | 4.09 | 4.40 | 8.43 | 11.52 |
| Y08-22 | Red | 12.82 | 0.46 | 21.41 | 23.88 | 13.45 | 9.73 | 3.84 | 4.57 | 8.70 | 13.70 |
| HB-5 | Red-orange | 4.03 | 0.37 | 18.15 | 20.52 | 14.58 | 9.71 | 3.98 | 3.41 | 6.71 | 8.77 |
| Y03-10 | Red | 9.62 | 0.62 | 24.60 | 25.51 | 13.37 | 9.63 | 2.53 | 4.79 | 6.45 | 10.04 |
| K2 | Yellow | 6.58 | 0.37 | 20.41 | 23.71 | 12.88 | 9.60 | 3.76 | 3.55 | 6.06 | 11.14 |
| 09-03 | Red | 4.50 | 0.32 | 15.41 | 21.75 | 11.35 | 9.58 | 2.37 | 3.41 | 6.92 | 10.51 |
| 10-06 | Yellow | 4.15 | 0.33 | 16.52 | 19.18 | 16.23 | 9.57 | 3.79 | 4.35 | 8.11 | 13.69 |
| 3-5-1-14 | Red | 8.16 | 0.50 | 20.16 | 24.59 | 13.06 | 9.40 | 3.92 | 3.95 | 7.04 | 10.21 |
| 15-40 | Yellow | 8.39 | 0.57 | 22.84 | 25.36 | 14.87 | 9.39 | 2.31 | 3.64 | 6.49 | 9.92 |
| DG-7 | Red | 3.34 | 0.33 | 17.90 | 18.47 | 12.34 | 9.33 | 3.89 | 3.86 | 7.78 | 10.46 |
| JO1H | Red | 2.86 | 0.25 | 16.80 | 17.98 | 9.08 | 9.32 | 2.42 | 5.12 | 6.55 | 11.03 |
| F3-1 | Red-orange | 2.57 | 0.24 | 14.96 | 15.96 | 17.84 | 9.27 | 3.29 | 4.35 | 6.47 | 10.29 |
| 09-38-1 | Red | 6.93 | 0.50 | 21.91 | 23.63 | 12.90 | 9.21 | 3.10 | 4.41 | 6.93 | 11.65 |
| J-2 | Yellow | 3.37 | 0.33 | 15.97 | 18.46 | 11.93 | 9.18 | 3.40 | 4.60 | 7.45 | 10.96 |
| DG-4-1 | Yellow | 3.24 | 0.26 | 16.09 | 18.72 | 15.46 | 9.14 | 3.59 | 3.62 | 6.59 | 10.75 |
| PZBG | Yellow | 10.36 | 0.60 | 25.04 | 28.09 | 11.17 | 9.10 | 3.59 | 3.51 | 7.54 | 11.59 |
| Ft3-1-2 | Red | 5.59 | 0.51 | 18.55 | 21.27 | 14.98 | 9.09 | 3.37 | 3.84 | 6.95 | 10.50 |
| S-D-2 | Red | 6.55 | 0.54 | 19.57 | 23.01 | 11.94 | 9.02 | 3.57 | 4.75 | 6.66 | 9.91 |
| 15-01 | Red-orange | 5.15 | 0.49 | 19.59 | 21.02 | 14.98 | 8.85 | 3.52 | 4.33 | 6.80 | 10.04 |
| N15-42 | Red | 5.44 | 0.33 | 19.31 | 22.92 | 15.17 | 8.76 | 3.25 | 3.19 | 6.87 | 10.77 |
| TXG | Red | 3.09 | 0.33 | 17.57 | 16.80 | 14.63 | 8.74 | 3.50 | 3.23 | 6.24 | 11.21 |
| SGX | Red | 9.37 | 0.66 | 21.75 | 25.39 | 10.53 | 8.67 | 3.74 | 4.91 | 7.76 | 12.17 |
| 1-17-2 | Red | 8.80 | 0.61 | 25.74 | 24.10 | 17.53 | 8.64 | 3.64 | 4.58 | 7.01 | 11.27 |
| 11-07 | Dark red | 2.84 | 0.27 | 14.79 | 17.36 | 16.25 | 8.53 | 3.51 | 3.44 | 7.35 | 9.86 |
| M19-4 | Red | 2.25 | 0.24 | 15.67 | 15.56 | 14.80 | 8.50 | 3.53 | 4.57 | 6.43 | 9.84 |
| Ft-4 | Red | 4.63 | 0.29 | 18.14 | 20.35 | 20.64 | 8.42 | 3.40 | 4.14 | 7.35 | 10.19 |
| GS-2 | Red | 9.38 | 0.93 | 22.96 | 25.03 | 11.20 | 8.41 | 3.48 | 4.23 | 7.30 | 12.33 |
| 628-1 | Red | 8.12 | 0.31 | 19.91 | 26.65 | 14.67 | 8.35 | 3.55 | 4.26 | 8.41 | 14.72 |
| Y06-22 | Red-orange | 4.05 | 0.39 | 16.91 | 20.03 | 17.24 | 8.23 | 3.16 | 4.05 | 6.57 | 10.11 |
| S-D-3 | Yellow | 7.82 | 0.49 | 21.44 | 25.57 | 10.08 | 8.09 | 3.60 | 3.51 | 6.25 | 8.68 |
| 16-11 | Red | 5.20 | 0.29 | 18.46 | 21.77 | 16.38 | 8.00 | 3.21 | 3.54 | 6.28 | 10.52 |
| 3-17-1 | Red | 3.37 | 0.28 | 18.32 | 16.46 | 17.03 | 7.98 | 3.06 | 2.84 | 7.00 | 9.34 |
| DS-1 | Light red | 2.21 | 0.32 | 15.21 | 16.13 | 11.22 | 7.90 | 3.65 | 3.94 | 6.88 | 9.84 |
| Y13-03 | Red-orange | 7.43 | 0.68 | 23.27 | 23.02 | 13.80 | 7.82 | 2.99 | 3.51 | 5.80 | 10.31 |
| 5B17-3 | Red | 4.61 | 0.30 | 17.67 | 19.13 | 13.58 | 7.68 | 3.04 | 3.52 | 6.66 | 12.10 |
| 5N17-2-1 | Red | 9.42 | 0.67 | 23.09 | 25.94 | 11.13 | 7.68 | 3.24 | 3.41 | 6.10 | 8.14 |
| 10-04 | Yellow | 3.47 | 0.33 | 17.29 | 17.65 | 12.75 | 7.64 | 2.95 | 3.15 | 4.68 | 7.61 |
| 34-1 | Red | 4.18 | 0.29 | 17.48 | 18.90 | 17.52 | 7.63 | 2.90 | 3.79 | 5.60 | 8.75 |
| Ft3-1-1 | Red | 6.35 | 0.56 | 20.89 | 22.82 | 12.46 | 7.61 | 3.00 | 3.58 | 5.51 | 9.97 |
| 03-35 | Red | 8.93 | 0.81 | 23.72 | 25.63 | 11.32 | 7.60 | 2.90 | 3.23 | 5.82 | 9.08 |
| 02-14 | Yellow | 9.36 | 0.57 | 23.53 | 25.79 | 10.85 | 7.48 | 2.91 | 3.39 | 5.43 | 8.54 |
| 13-05 | Red-orange | 6.90 | 0.53 | 21.13 | 23.36 | 10.40 | 7.46 | 2.74 | 3.62 | 5.80 | 8.35 |
| 15-10 | Red | 7.96 | 0.42 | 20.66 | 25.64 | 16.40 | 7.44 | 2.79 | 3.81 | 5.36 | 8.48 |
| M19-4-1 | Light red | 2.51 | 0.20 | 14.40 | 16.66 | 16.10 | 7.41 | 3.01 | 3.29 | 5.60 | 9.19 |
| 15-42 | Light red | 6.67 | 0.47 | 19.63 | 23.56 | 13.36 | 7.34 | 2.81 | 3.42 | 6.55 | 8.18 |
| 3-30-17-1 | Yellow | 6.95 | 0.38 | 20.37 | 23.41 | 14.60 | 7.29 | 2.60 | 2.50 | 4.69 | 7.04 |
| ZYOL | Dark red | 10.76 | 0.59 | 23.40 | 27.48 | 15.73 | 7.21 | 2.23 | 4.40 | 7.09 | 11.06 |
| XZ-1 | Red | 3.77 | 0.27 | 16.54 | 18.03 | 18.20 | 6.92 | 3.05 | 4.00 | 6.36 | 9.13 |
| 16-10M | Light red | 5.04 | 0.45 | 18.49 | 20.92 | 11.38 | 6.90 | 2.98 | 3.30 | 5.18 | 9.59 |
| HB-8 | Red | 2.73 | 0.28 | 15.55 | 16.94 | 11.50 | 6.81 | 1.58 | 2.85 | 5.12 | 8.30 |
| 09-38-2 | Red-orange | 6.83 | 0.32 | 20.68 | 23.52 | 16.13 | 6.71 | 2.56 | 3.03 | 4.60 | 7.74 |
| FDHG | Red | 9.32 | 0.60 | 22.32 | 25.59 | 11.48 | 6.70 | 2.40 | 2.65 | 4.56 | 7.30 |
| 19-05 | Yellow | 7.88 | 0.52 | 21.64 | 22.54 | 12.58 | 6.49 | 2.70 | 3.47 | 5.41 | 8.20 |
| Ft-1 | Red | 11.96 | 0.85 | 23.08 | 29.16 | 13.24 | 6.38 | 2.69 | 3.50 | 5.08 | 8.95 |
| 10-03 | Red | 9.40 | 0.34 | 22.35 | 24.57 | 12.45 | 6.27 | 1.44 | 3.64 | 4.24 | 6.90 |
| 3-4-4-20 | Red | 7.72 | 0.49 | 22.83 | 25.61 | 12.83 | 6.15 | 1.91 | 3.12 | 4.34 | 7.25 |
| LB-28 | Yellow | 6.26 | 0.43 | 19.00 | 23.58 | 14.03 | 6.08 | 1.90 | 3.18 | 4.29 | 7.34 |
| Y13-09 | Yellow | 5.15 | 0.35 | 17.24 | 21.29 | 16.88 | 6.08 | 2.20 | 3.06 | 4.83 | 7.48 |
| T-HB-4 | Red | 4.90 | 0.41 | 18.91 | 20.90 | 13.45 | 6.04 | 2.85 | 3.01 | 4.65 | 6.78 |
| S-D-1 | Red | 15.61 | 0.93 | 24.90 | 31.32 | 13.74 | 6.02 | 2.36 | 3.00 | 4.39 | 6.79 |
| 15-51 | Red | 6.55 | 0.52 | 20.41 | 24.52 | 12.14 | 5.99 | 2.58 | 3.15 | 5.91 | 7.62 |
| 15-11 | Red | 6.87 | 0.41 | 19.90 | 23.70 | 13.40 | 5.91 | 2.52 | 3.79 | 5.01 | 8.21 |
| 628 | Dark red | 1.68 | 0.21 | 13.96 | 14.32 | 14.90 | 5.85 | 2.73 | 2.92 | 5.73 | 7.55 |
| 08-24 | Red | 9.33 | 0.81 | 19.69 | 25.65 | 13.60 | 5.73 | 2.13 | 2.47 | 3.82 | 7.30 |
| HB-6 | Yellow | 8.40 | 0.45 | 24.53 | 26.32 | 13.30 | 5.65 | 2.43 | 3.00 | 4.32 | 6.55 |
| T1-1-17-1 | Red | 13.37 | 0.60 | 25.37 | 31.17 | 13.63 | 5.60 | 1.70 | 3.46 | 5.01 | 7.49 |
| 3-3-4-5 | Red | 7.50 | 0.45 | 20.48 | 24.24 | 13.50 | 5.33 | 1.91 | 3.12 | 4.34 | 7.25 |
| T17-1 | Red | 7.67 | 0.47 | 20.26 | 24.36 | 10.98 | 5.07 | 2.02 | 2.62 | 4.00 | 7.83 |
| ZS-3 | Red | 7.63 | 0.46 | 19.52 | 25.64 | 11.04 | 3.90 | 1.65 | 2.11 | 3.25 | 4.68 |
