# Supplementary material for: Fruit quality and antioxidant potential of Prunus humilis Bunge accessions
Source: PLoS One. 2020 Dec 30;15(12):e0244445. doi: 10.1371/journal.pone.0244445 (PMC7773198; doi:10.1371/journal.pone.0244445)
Supplement: Attachment — Submitted filename: response to reviewers.doc [file pone.0244445.s005.doc]

The authors show lack of familiarity with the study species since they did not perform literature survey in order to compare the results they have obtained with the ones already published.

**Reply: modifications have been made as required and marked in blue font in the text, we have complemented the manuscript content.**

Main title: "accessions", not "germplasms" (and further in the text). Germplasm is a collective noun and cannot read in plural.

**Reply: modifications have been made in the full manuscript as required and marked in blue font in the text.**

Many data are not provided in the M&M section, such as info on the accessions studied. Inconsistencies in measuring process are also notified by the reviewers, while the data visualization can be improved.

**Reply: data has been provided as required, shown in Supplement 3.**

The whole text has to be thoroughly reorganized in order to keep the flow and improve readability. Some parts (particularly Introduction and Discussion sections) need to be fully rewritten as suggested in the reviewers' comments.

**Reply: modifications have been made as required and marked in blue font in the text, we have revised and complemented the manuscript content in Introduction and Discussion sections.**

Reviewer #1:

1. The aim of the study is interesting, but the quality of the manuscript is poor.
   The introduction is superficial, and more background is needed in order to understand the overall purpose of this study: authors have not considered the presence in literature of many papers dealing with antioxidant metabolites and antioxidant properties in cherry fruits.

**Reply: modifications have been made as required and marked in blue font in the text, we have revised and complemented the manuscript content in Introduction.**

2. Authors deal with 137 C. humilis germplasms; however, they do not include any detail of the species or accessions in the Material and Methods section. A more detailed explanation of plant material must be included in this section, and a list of the 137 plant accessions should be added as supplementary table.

**Reply: data has been provided as required, shown in Supplement 1.**

3. “Field management included conventional irrigation and fertilization methods”. This is an international journal and the conventional specifications for cultivation in China may be different from those of other Nations. Therefore, everything must be written in detail or referred to another publication in which the cultivation method has been already explained in detail.

**Reply: modifications have been made as required and marked in blue font in the text, we have revised and complemented the manuscript content in Materials and Methods.**

4. “Total phenol content (TPC) was determined by Folin-Ciocalteu colorimetry [17]. A certain volume of extracted solution”: what do you mean with “a certain volume”? This is a scientific article, everything must be correctly defined.

**Reply: modifications have been made as required and marked in blue font in the text, we have revised and complemented the manuscript content in Materials and Methods.**

5. “Following the method of Zhang [18], we added 2.8 mL of 0.1 mM DPPH to 0.2 mL of sample diluent.”: What do authors mean with “sample diluent”? Methanolic extract?

**Reply: modifications have been made as required and marked in blue font in the text, we have revised and complemented the manuscript content in Materials and Methods.**

6. About the FRAP assay: “For the blank, the diluent was replaced with 40% (v/v) methanol.”: Do authors mean “40% (v/v) acidified methanol”?

**Reply: modifications have been made as required and marked in blue font in the text, we have revised and complemented the manuscript content in Materials and Methods.**

7. The Trolox assay must be better specified.

**Reply: modifications have been made as required and marked in blue font in the text, we have revised and complemented the manuscript content in Materials and Methods.**

8. About the cluster analysis, I do not understand why the heat map analysis has been performed using only one parameter and not including all the analyses parameters. An analysis like heat map or PCA represents a useful tool for the analysis of multivariate data that could offer an overview of the results and visualize the real differences between different accessions.

**Reply: modifications have been made as required and marked in blue font in the text, we have revised and complemented the manuscript content in Results.**

In this study, after determined and clustering the flavonoid content of *Cerasus humilis* accessions was systematically classified, and the boundary between the flavonoid content and the flavonoid content of *Cerasus humilis* fruit was defined. The distribution range of flavonoids in *Cerasus humilis* fruit could be seen. At the same time, the joint analysis with the fruit peel color was carried out in the follow-up in order to compare the relationship between flavonoid content and peel color. So the heat map analysis has been performed using only one parameter and not including all the analyses parameters.

9. The discussion is too general and not very focused on the data obtained. Probably the fact that the entire set of data was not subjected to a multivariate analysis (heay map or PCA) did not allow the authors to fully understand the differences between the accessions analysed and the potential of their own study.

**Reply: modifications have been made as required and marked in blue font in the text, we have revised and complemented the manuscript content in Results.**

Reviewer #2:
1. Authors point out that they aimed to elucidate flavonoid biosynthesis and I do not agree with them. In my opinion they have studied the fruit quality, mainly related to flavonoids, but not their biosynthesis.

**Reply: modifications have been made as required and marked in blue font in the text.**

2. A list of abbreviations will be very helpful.

**Reply: data has been provided as required, shown in Supplement 2.**

3. Define the abbreviations at Table 6 legend.

**Reply: modifications have been made as required and marked in blue font in the text, we have revised it at table 6.**

4. Have you studied other anthocyanidins apart from Cyanidin-3-gluscoside? Red fruits and mainly dark red fruits should have large quantities of these compounds.

**Reply: the specific answer is as follows:**

In our early research, the metabonomics of 19-04 (red) were determined, and 20 components of anthocyanins were detected (Peonidin O-hexoside, Pelargonidin 3-O-malonylhexoside, Peonidin, Cyanidin O-syringic acid, Cyanidin O-acetylhexoside, Cyanidin 3-O-glucoside, Delphinidin, Malvidin 3-O-galactoside, Malvidin 3-O-glucoside, Delphinidin 3-O-glucoside, Cyanidin 3-O-rutinoside, Cyanidin 3,5-O-diglucoside, Malvidin 3,5-diglucoside, Pelargonin, Petunidin 3-O-glucoside, Pelargonidin 3-O-beta-D-glucoside, Cyanidin, Cyanidin 3-O-galactoside, Peonidin 3-O-glucoside chloride and Malvidin 3-acetyl-5-diglucoside). After absolute quantitative analysis by UHPLC, the content of Cyanidin 3-O-glucoside accounted for more than 63% of the total content. Therefore, we believe that Cyanidin 3-O-glucoside is an important anthocyanin in *Cerasus humilis* fruit.

**At the same time, the manuscript has also been revised accordingly in Discussion.**

5. Discussion is comprehensive and readable but can be improved. A deeper discussion relating data is needed.

**Reply: modifications have been made as required and marked in blue font in the text, we have revised and complemented the manuscript content in Discussion.**

6. May be a Principal component analysis of data should be interesting.

**Reply: modifications have been made as required and marked in blue font in the text, we have revised and complemented the manuscript content in Results.**
