# Supplementary material for: Fruit quality and antioxidant potential of Prunus humilis Bunge accessions
Source: PLoS One. 2020 Dec 30;15(12):e0244445. doi: 10.1371/journal.pone.0244445 (PMC7773198; doi:10.1371/journal.pone.0244445)
Supplement: Attachment — Submitted filename: response to reviewers.doc [file pone.0244445.s006.doc]

Thank you for your comments again,and I will list my changes as below:

1. The authors show lack of familiarity with the study species since they did not perform literature survey in order to compare the results they have obtained with the ones already published.

**Reply:revised have been made as required, and the content as following:**page 25, line 345, add the contents after(1.2–15.61 g; average: 6.13 g), *Cerasus humilis* in Hebei province were measured and found that the largest weight was 10.11 g and the smallest weight was 3.67 g [22],we found that our *C. humilis* weight varies more widely. It is well known that fruit weight in differernt area, but under similar cultivation conditions in the same area also produce differernt weights, it mean that it has a close relationship between the fruit weight and accessions. **And** at page 25, line 349 add the contents after 7.04%–20.64%; compared with the seven *C. humilis* fruit in Hebei province (7.43%-15.90%), our fruits are with high soluble solid content. **And** at page 26, line 367 add the contents after 10.58 mg/g.The polyphenol content of 28 different genotypes in Liaoning Province were determined, and a total of 31 polyphenols were detected, included 17 flavonols and 1 flavone, among them the Qu3Ara (quercetin 3-O-arabinoside) was the dominant component, and content tanging from 7.44 to 37.89 mg/100g DW [21]. As we know red fruits and mainly dark red fruits should have large quantities of these anthocyanidins, in our early research, the metabonomics of 19-04 (red) were determined, and 20 components of anthocyanins were detected. After absolute quantitative analysis by UHPLC, the content of Cyanidin 3-O-glucoside accounted for more than 63% of the total content.**And** at page 26, line 376 add the contents after antioxidant indices.About the research of sweet cherry, the fruits were characterized by a higher antioxidant activity, DPPH radical scavenging activity was about 10 mmol TE/100g DW[41], it is strong positive correlations between fruit phenolic content [18]. The antioxidation test about *C. humilis* in Liaoning Province found that the FRAP free radical scavenging capacity (9.52–29.44mg/g DW) was the highest and the ABTS free radical scavenging capacity was the lowest (3.40–12.88mg/g DW)[21]. But in our study, ABTS free radical scavenging capacity (4.68–24.23mg/g FW)was the highest, the difference may be due to fresh and dry samples, the flavonoids changed during drying. It may cause the difference of free radical scavenging ability.

2. Main title: "accessions", not "germplasms" (and further in the text). Germplasm is a collective noun and cannot read in plural.

**Reply:revised have been made in the full manuscript as required.**

3. Many data are not provided in the M&M section, such as info on the accessions studied. Inconsistencies in measuring process are also notified by the reviewers, while the data visualization can be improved.

**Reply:data about accessions** **has been provided as required, shown in Supplement 3.**

4. The whole text has to be thoroughly reorganized in order to keep the flow and improve readability. Some parts (particularly Introduction and Discussion sections) need to be fully rewritten as suggested in the reviewers' comments.

**Reply:revised have been made as required and marked in blue font in the text, Introduction at page 2, line 40 ,we revised the content as below,** Chinese dwarf cherry (*Cerasus humilis* (Bge.) Sok) (Rosaceae) is a small deciduous shrub [1]. It is like peach, plum and apricot, which is an ancient tree species in China with a cultivation history dated back to around 3000 years [2]. It is grown in the north of China recorded about 13 provinces, i.e., Shanxi, Hebei, Liaoning provinces, and so on [3]. The root of *C. humilis* is well developed and has strong ecological adaptability [4]. *C. humilis* has the characteristics of drought, barren tolerance and soil-water conservation. It has been applied by the forestry bureau in the Sand Control Project around Beijing and Tianjin area and the forest ecological project of three North shelterbelt [5]. *C. humilis* are known as ‘calcium fruit’ in China due to the high calcium content [6]. **And** at page 2, line 43,after the content ubstances [2], we add the content It can be consumed fresh or used in wine, juice, jam, and several other products. **And** at page 3, line 56, after the content many human diseases [12], we add the content An increasing incidence of non-communicable diseases, all closely associated with oxidative stress, motivates scientists to look for natural methods of disease prevention. Fruits are an important component of traditional food, but are also central to healthy diets of modern urban population [17], including *Cerasus humilis*, represent potent sources of bioactive compounds, with strong health-promoting and disease-preventive activities [18]. These compounds present a strong antioxidant capacity and also can scavenge free radicals. **And** at page 4, from line 68 to 72, we revised the content to In the early research, some researchers have measured and analyzed the fruit quality of *C. humilis* accessions. Total flavonoid content and radical scavenging activity of 16 *C. humilis* genotypes were determined [3]. The polyphenol compounds of 28 and 13 different genotypes of *C. humilis* in Liaoning province [21] and Beijing city [2] were systematically characterized, respectively. Seven varieties of *C. humilis* were evaluated [22]. Although the fruit quality of *C. humilis* has been evaluated, the number of assessions was small. To the best of our knowledge, this is the first study to assess the fruits derived from *C. humilis* accessions on a large scale. We selected 137 *C. humilis* accessions and analyzed the basic characteristics, bioactive compounds, and antioxidant capacities of their fruit to evaluate the fruit quality in *C. humilis* and identify cultivars that could provide superior human health benefits. **And** at page 4, from line 74, after the Moreover ,the content was delect. **And** at page 26, line 384 add the contents after *Rosa roxburghii* (chestnut rose) There are more than 9000 components of flavonoids widely existing in plants, according to the structure of flavonoids, we can classify them as flavones, flavanones, flavanols, isoflavones, flavonols, anthocyanidin and flavanonols. Each component has its own function, at the same time the difference in substance content resulted the difference in antioxidant activity. So there are differences in the ability on antioxidant indices in different species. It may be that there are more components in *C.humilis* fruit which have the ability of scavenging ABTS free radicals, so that the *C.humilis* fruit has a stronger ability of scavenging ABTS free radicals.

**And the revised answer about discussion was the same as the question 1.**

Reviewer #1:

5. The aim of the study is interesting, but the quality of the manuscript is poor.
   The introduction is superficial, and more background is needed in order to understand the overall purpose of this study: authors have not considered the presence in literature of many papers dealing with antioxidant metabolites and antioxidant properties in cherry fruits.

**Reply:revised have been made as required, a**nd t**he content as following:page 3, line 56, add the contents after many human diseases [12],** An increasing incidence of non-communicable diseases, all closely associated with oxidative stress, motivates scientists to look for natural methods of disease prevention. Fruits are an important component of traditional food, but are also central to healthy diets of modern urban population [17], including *Cerasus humilis*, represent potent sources of bioactive compounds, with strong health-promoting and disease-preventive activities [18]. These compounds present a strong antioxidant capacity and also can scavenge free radicals.

6. Authors deal with 137 *C. humilis* germplasms; however, they do not include any detail of the species or accessions in the Material and Methods section. A more detailed explanation of plant material must be included in this section, and a list of the 137 plant accessions should be added as supplementary table.

**Reply: data has been provided as required, shown in Supplement 1.**

7. “Field management included conventional irrigation and fertilization methods”. This is an international journal and the conventional specifications for cultivation in China may be different from those of other Nations. Therefore, everything must be written in detail or referred to another publication in which the cultivation method has been already explained in detail.

**Reply:revised have been made as required and marked in blue font in the text, at page 4, line 83, we add the content after** f**ertilization methods**.The growth period was fertilized twice a year, once before germination in spring and once after winter, 40kg nitrogen fertilizer per 667m^2^, mainly by hole application. From May to August watering once or twice a month, in the early November enough water was irrigated for overwintering.

8. “Total phenol content (TPC) was determined by Folin-Ciocalteu colorimetry [17]. A certain volume of extracted solution”: what do you mean with “a certain volume”? This is a scientific article, everything must be correctly defined.

**Reply:we revised the content about A certain volume of to** First, 0.2 mL of the **at page 6, line 131.**

9. “Following the method of Zhang [18], we added 2.8 mL of 0.1 mM DPPH to 0.2 mL of sample diluent.”: What do authors mean with “sample diluent”? Methanolic extract?

**Reply:we revised the content about sample diluent to** extracted solution. **at page 7, line 142, and** diluent was changed to extracted solution at page 7, line 143.

10. About the FRAP assay: “For the blank, the diluent was replaced with 40% (v/v) methanol.”: Do authors mean “40% (v/v) acidified methanol”?

**Reply:we revised the content about sample diluent to** extracted solution. **at page 7, line 157, and** diluent was changed to extracted solution at page 8, line 158.

11. The Trolox assay must be better specified.

**Reply:we revised the title to Trolox evaluate,And add the content after the** activity [19], The results of DPPH, FRAP and ABTS were expressed as mg of Trolox equivalents (TE) per g of fruit (on a fresh weight basis).

12. About the cluster analysis, I do not understand why the heat map analysis has been performed using only one parameter and not including all the analyses parameters. An analysis like heat map or PCA represents a useful tool for the analysis of multivariate data that could offer an overview of the results and visualize the real differences between different accessions.

**Reply:** In this study, after determined and clustering the flavonoid content of *Cerasus humilis* accessions was systematically classified, and the boundary between the flavonoid content and the flavonoid content of *Cerasus humilis* fruit was defined. The distribution range of flavonoids in *Cerasus humilis* fruit could be seen. At the same time, the joint analysis with the fruit peel color was carried out in the follow-up in order to compare the relationship between flavonoid content and peel color. So the heat map analysis has been performed using only one parameter and not including all the analyses parameters. **And we add the content of PCA at page 24, line 336, as following:**

**Principal component analysis**

The PCA results identified two components that explained 86.12% of the total variation in fruit quality among the 62 *Cerasus humilis* accessions (Table 7). The first principal component (Prin1) contributed 52.56% of the total variation, large positive values were associated with total flavonoid content and ABTS, suggesting that these two indices greatly contributed to Prin1. The second principal component (Prin2) contributed 33.56% of the total variation, large positive values were associated with single fruit weight,vertical and horizontal diameter, suggesting that these three indices greatly contributed to Prin2.

Scatterplots from the PCA based on the fruit quality showed that 10-02, 3-17-2, 3-17-4, and JD1-6-7-37 belonged in the first group (Fig 8), characterized by high TFC, ABTS and large fruit. Other four group also had different characteristics. These data suggest that crossing within groups maybe more efficient for directed breeding.

13. The discussion is too general and not very focused on the data obtained. Probably the fact that the entire set of data was not subjected to a multivariate analysis (heay map or PCA) did not allow the authors to fully understand the differences between the accessions analysed and the potential of their own study.

**Reply: the revised answer about discussion was the same as the question 1, And** at page 26, line 384 add the contents after *Rosa roxburghii* (chestnut rose) There are more than 9000 components of flavonoids widely existing in plants, according to the structure of flavonoids, we can classify them as flavones, flavanones, flavanols, isoflavones, flavonols, anthocyanidin and flavanonols. Each component has its own function, at the same time the difference in substance content resulted the difference in antioxidant activity. So there are differences in the ability on antioxidant indices in different species. It may be that there are more components in *C.humilis* fruit which have the ability of scavenging ABTS free radicals, so that the *C.humilis* fruit has a stronger ability of scavenging ABTS free radicals. **and before the Conclusions at the page 28, line 413, we add the content** The principal component analysis results identified two components included TFC, ABTS free radical scavenging activity, single fruit weight,vertical and horizontal diameter. Based on these five indices, we grouped different accessions into five categories with different characteristics. 10-02, 3-17-2, 3-17-4 and JD1-6-7-37 are rich in TFC and strong antioxidant activity that might be more widely used in general population and the food industry as a source of bioactive to improve human health.

Reviewer #2:

14. Authors point out that they aimed to elucidate flavonoid biosynthesis and I do not agree with them. In my opinion they have studied the fruit quality, mainly related to flavonoids, but not their biosynthesis.

**Reply:we delect the content related to** biosynthesis at page 4,from line 70 to 75, and page 25, line 341.

15. A list of abbreviations will be very helpful.

**Reply: data has been provided as required, shown in Supplement 2.**

16. Define the abbreviations at Table 6 legend.

**Reply: revised have been made as required and marked in blue font in the text, we have revised it at table 6 at page 24, line 316.**

17. Have you studied other anthocyanidins apart from Cyanidin-3-gluscoside? Red fruits and mainly dark red fruits should have large quantities of these compounds.

**Reply: the specific answer is as follows:**

In our early research, the metabonomics of 19-04 (red) were determined, and 20 components of anthocyanins were detected (Peonidin O-hexoside, Pelargonidin 3-O-malonylhexoside, Peonidin, Cyanidin O-syringic acid, Cyanidin O-acetylhexoside, Cyanidin 3-O-glucoside, Delphinidin, Malvidin 3-O-galactoside, Malvidin 3-O-glucoside, Delphinidin 3-O-glucoside, Cyanidin 3-O-rutinoside, Cyanidin 3,5-O-diglucoside, Malvidin 3,5-diglucoside, Pelargonin, Petunidin 3-O-glucoside, Pelargonidin 3-O-beta-D-glucoside, Cyanidin, Cyanidin 3-O-galactoside, Peonidin 3-O-glucoside chloride and Malvidin 3-acetyl-5-diglucoside). After absolute quantitative analysis by UHPLC, the content of Cyanidin 3-O-glucoside accounted for more than 63% of the total content. Therefore, we believe that Cyanidin 3-O-glucoside is an important anthocyanin in *Cerasus humilis* fruit.

**At the same time, the manuscript has also been revised accordingly in Discussion, at revised manuscript page 30,line 445,** As we know red fruits and mainly dark red fruits should have large quantities of these anthocyanidins, in our early research, the metabonomics of 19-04 (red) were determined, and 20 components of anthocyanins were detected. After absolute quantitative analysis by UHPLC, the content of Cyanidin 3-O-glucoside accounted for more than 63% of the total content.

18. Discussion is comprehensive and readable but can be improved. A deeper discussion relating data is needed.

**Reply:the revised answer about discussion was the same as the question 13.**

19. May be a Principal component analysis of data should be interesting.

**Reply:the revised answer about discussion was the same as the question 12.**
