# Supplementary material for: Fruit quality and antioxidant potential of Prunus humilis Bunge accessions
Source: PLoS One. 2020 Dec 30;15(12):e0244445. doi: 10.1371/journal.pone.0244445 (PMC7773198; doi:10.1371/journal.pone.0244445)
Supplement: Attachment — Submitted filename: Response to reviewer.docx [file pone.0244445.s007.docx]

Thank you for your comments again,and I will list my changes as below:

1. The authors did not respond to this important issue through two rounds of the review process. To make myself more clear: *Prunus humilis* Bunge is the internationally accepted botanical name of the study species and *Cerasus humilis* (Bunge) S.Ya.Sokolov is a synonym. Therefore, P. humilis should be used through the text, while its synonym can be written in parenthesis first time it is mentioned in the Introduction section.

**Reply**: *Cerasus humilis* has been replaced by *Prunus humilis* through the text,and marked in **blue** font.

2. The authors show lack of familiarity with the study species since they did not perform literature survey in order to compare the results they have obtained with the ones already published.

**Reply:** We have compared the references Free Radical Scavenging Activities of Polyphenols from *Prunus humilis* Bge Fruit.and Analysis of polyphenols composition and antioxidant activity assessment of Chinese dwarf cherry (*Cerasus humilis*(bge.) Sok.), and we have discussed the other references,example [2] , [3] , [6] , [7] and [22] in the DISCUSSION.

1) Page 30, lie 455, after the cells, we add the content of **Free radicals are unstable and therefore have a short life span. It is an important method to evaluate the antioxidant activity of antioxidants by studying their scavenging ability. Due to the difference** n**of chemical property and reaction environment of different free radicals, it is important to select suitable free radicals for evaluating the biological activity and structure-activity relationship of free radical scavengers** [1]**.**
2) We compared the reference [21] (Analysis of polyphenols composition and antioxidant activity assessment of Chinese dwarf cherry (*Cerasus humilis*(bge.) Sok.)) at **page 29, line 428-431**.
3) Also we compared the reference [22] at page 28,line 398-400. ***Prunus humilis* fruits in Hebei province were measured, and it was found that the largest weight was 10.11 g and the smallest weight was 3.67 g [22]; however, we found that our *P. humilis* weight varied more widely. It is well known that fruit weight varies between areas, but under similar cultivation conditions in the same area, *P. humilis* also produces fruit of different weights. This finding means that fruit weight depends on accessions and not geographic areas. Moreover, the genetic diversity in our study was rich. Hence, these accessions furnish abundant material for the selection and breeding of fruits of the required size. SSC directly determines fruit flavor; usually, the higher the soluble solid content, the better the flavor and quality. Thus, the selection of *P. humilis* with high SSC is an important breeding target. In this study, the SSC in *P. humilis* fruit was 7.04%–20.64%. Compared with the seven *P. humilis* fruits in Hebei province (7.43%-15.90%), our fruits have higher soluble solid content.**
3. The language is still one of the major concerns. Some of them are stated in the reviewers' reports but I strongly recommend revising the manuscript by a professional editing agency.

**Reply**: About our English editing,first, thanks for the editing by Wiley Editing Services. And we have maked some changes as below and has marked in blue font in the manuscript:

1) Page 2, line 33, among was changed with **between**.
2) Page 2, line 39, before the vertical add the content of **and**.
3) Page 2, line 40,component was changed with **components**, and the accessions was change with **accession**.
4) Page 2, line 40 to line 42,the 10-02, 3-17-2, 3-17-4, and JD1-6-7-37 characterized by high TFC, ABTS and large fruit, out results will aid in breeding and functional food processing of the *Cerasus humilis.*was change with **Accessions 10-02, 3-17-2, 3-17-4, and JD1-6-7-37 were characterized by high TFC, ABTS, and large fruit. We believe that our results will aid in the breeding and functional food processing of *Prunus humilis.***
5) Page 3, line 47, the content of It is like after [1]was change with **Like**,and Page 2, line 48,the content of dated was change with **dates**.
6) Page 3, line 51-54,the content of *C. humilis* has the characteristics of drought, barren tolerance and soil-water conservation. It has been applied by the forestry bureau in the Sand Control Project around Beijing and Tianjin area and the forest ecological project of three North shelterbelt was changed with ***Prunus humilis* can grow on barren land, withstand drought, and conserve soil water. It has been used by the Forestry Bureau in the Sand Control Project around Beijing and Tianjin areas and in the Three-North Shelter Forest Program.**
7) Page 3, line 54, the content of *C. humilis* was changed with **The fruits of *P. humilis.***
8) Page 3, line 57, the content of it after [7] was changed with **They**.
9) Page 4, line 72, the content of motivates was changed with **is motivating.**
10) Page 4, line 73, the content of methods of disease prevention was changed with **disease prevention method.**
11) Page 4, line 74-75, the content of but are also central to healthy diets of modern urban population was chenged with **and are also essential items in a healthy diet for the modern urban population.** And including *Cerasus humilis* represent was changed with **Moreover, in this view fruits such as *Prunus humilis*, represents.**
12) Page 4, line 76, preventive was changed with **preventing**.
13) Page 4, line 77, strong was changed with **have**.
14) Page 4, line 89, the early research, some was changed with **previous studies.**
15) Page 4, line 91, were was changed with **have been.**
16) Page 4, line 93, were was changed with **have been.**
17) Page 5, line 95, assessions was changed with **accessions.**
18) Page 5, line 97, basic was changed with **essential.**
19) Page 5, line 98, after the of ,add the content of **the.**
20) Page 5, line 107-112,the content of The growth period was fertilized twice a year, once before germination in spring and once after winter, 40kg nitrogen fertilizer per 667m^2^, mainly by hole application. From May to August watering once or twice a month, in the early November enough water was irrigated for overwintering. Between June and October, 137 fully ripe *C. humilis* accessions (S1 Table) were harvested in 2018 and 2019 was changed with **During the growth period, the plants were fertilized twice a year, once before germination in spring and once after winter. The amount was 40 kg of nitrogen fertilizer per 667m^2^, mainly by hole application. From May to August, the plants were watered once or twice a month, and, in early November the plants were irrigated with enough water for wintering. Between June and October, in 2018 and 2019, 137 fully ripe *P. humilis* accessions (S1 Table) were harvested.**
21) Page 10, line 215, The abbreviation are detailed in S2 Table was changed with **The abbreviations are detailed in Table S2.**
22) Page 10, line 219, the content of two year was changed with **two-year.**
23) Page 10, line 225, after the between, we add the content of **the.**
24) Page 25, line 344,the indicates was changed with **indicate.**
25) Page 26, line 369, after the variation, we add the content of **and**.
26) Page 26, line 371, after the indices, the **greatly** **was delete**, and after the contributed, we add the content of **significantly.**
27) Page 26, line 377, Other four group also was changed with **The other four groups.**
28) Page 29, line 427, were was changed with **was.** And included was changed with **including.**
29) Page 29, line 429, tanging was changed with **ranging.**
30) Page 29, line 431, between significantly positively, we add the content of **and**.
31) Page 29, line 437, that was changed with **which**.
32) Page 30, line 456, About the research of was changed with **Regarding**.

33)Page 30, line 458, it is strong positive correlations between was changed with **and that was strongly and positively correlated with**.

34) Page 30, line 459, the about was changed with **on**.

35) Page 30, line 462, But was changed with **However**.

36) Page 30, line 464,after the content of samples, we add the content of **and**.and before the changed ,we add the content of **may have**.and it may was changed with **This effect could**.

37) Page 30, line 465, the was changed with **a**.and of was changed with **in**.

38) Page 31, line 473, after the [44], we add the content of **More than 9000 flavonoid components occur widely in plants. According to their structure, these flavonoids are classified as flavones, flavanones, flavanols, isoflavones, flavonols, anthocyanidin, and flavanonols. Each component has its own function, and at the same time, the difference in flavonoid content causes differences in antioxidant activity. So different species differ in antioxidant capacity. In particular, *P*. *humilis* has a stronger ability of scavenging ABTS free radicals probably because contain a higher number of different antioxidant metabolites.**

39) Page 31, line 491, includded was changed with **including.**

40) Page 32, line 495, might changed with **could**.

41) Page 32, line 496, before the general ,add **the**.and bioactive changed with **bioactives**.

42) **We have changed the reference 1.**
