# Supplementary material for: Fruit quality and antioxidant potential of Prunus humilis Bunge accessions
Source: PLoS One. 2020 Dec 30;15(12):e0244445. doi: 10.1371/journal.pone.0244445 (PMC7773198; doi:10.1371/journal.pone.0244445)
Supplement: Attachment — Submitted filename: response to reviewers.docx [file pone.0244445.s008.docx]

Dear editor:

Thank you for your understand and support!

And thanks for your comments again,and I will list my changes as below:

1. As I commented in the previous review round, full scientific name of the studied species must stand. Hence, "Prunus humilis Bunge" must stand in the main title (without quotation marks), and in the first mention in the Introduction section. There (in the Introduction section), its synonym "Cerasus humilis (Bunge) S.Ya.Sokolov" should be provided in parenthesis (in brackets) of the actual scientific name.

Reply: In the title, we delete the Chinese dwarf cherry, the last title is Fruit quality and antioxidant potential of *Prunus humilis* Bunge accessions.

And in the Introduction section, Chinese dwarf cherry was replaced by *Prunus humilis* Bunge (*Cerasus humilis* (Bunge) S.Ya.Sokolov).

2. One more most important point: Figure 1, Figure 2 and Figure 6 are of very poor quality.Labels in Fig.1 and Fig.6 are not visible. Resolution of Fig.2 is too low.

**Reply:** We have update the figure 1, 2 and 6. and we have upload the figures in the Revise Submission.

Thanks for your understand and support very much again!
