# Supplementary material for: Fruit quality and antioxidant potential of Prunus humilis Bunge accessions
Source: PLoS One. 2020 Dec 30;15(12):e0244445. doi: 10.1371/journal.pone.0244445 (PMC7773198; doi:10.1371/journal.pone.0244445)
Supplement: Attachment — Submitted filename: response to reviewers.docx [file pone.0244445.s009.docx]

Dear editor:

Thank you for your understand and support again!

And thanks for your comments again,and I will list my changes as below:

1. Authors did not make visible labels in Figure 2. Since the information presented there is hardly readable, I suggest moving the Figure 2 into the Supplementary material. Figures 3 - 6 must be renamed consecutively and their references in the text should be updated too.

Reply:we moved the Figure 2 into the supplement as the Figure S1.and we also updated the figures 3-8 to 2-7 accordingly.

Thanks for your understand and support very much again!
